# miRNA-221: A Potential Biomarker of Progressive Liver Injury in Chronic Liver Disease (CLD) due to Hepatitis B Virus (HBV) and Nonalcoholic Fatty Liver Disease (NAFLD)

**DOI:** 10.1155/2024/4221368

**Published:** 2024-08-16

**Authors:** Parthana Rani Sutradhar, Nahida Sultana, Afzalun Nessa

**Affiliations:** ^1^ Department of Microbiology Sher-E-Bangla Medical College (SBMC), Barishal, Bangladesh; ^2^ Department of Virology Bangabandhu Sheikh Mujib Medical University (BSMMU), Dhaka, Bangladesh

**Keywords:** chronic liver disease, fibrosis, hepatitis B virus, microRNA, NAFLD

## Abstract

**Background:** Early detection of progressive liver damage in chronic liver disease (CLD) patients is crucial for better treatment response. Several studies have shown the association of microRNA (miRNA) in the progression of CLD in regulating cell proliferation, fibrosis, and apoptosis as well as in carcinogenesis.

**Objectives:** The study was aimed at determining the expression of miRNA-221 among different stages of fibrosis in CLD patients due to hepatitis B virus (HBV) and nonalcoholic fatty liver disease (NAFLD) and thus evaluate its role as an early biomarker in CLD.

**Methods:** A total of 100 participants (75 CLD patients and 25 healthy control) were recruited in this cross-sectional study and divided into four groups, of which 25 as healthy control, 25 in CLD without fibrosis, 25 were CLD with fibrosis, and 25 were CLD with cirrhosis. Total RNA was extracted from plasma followed by cDNA synthesis, and finally, the expression of miRNA-221 was analyzed for its diagnostic potential as a single biomarker using the qRT-PCR method.

**Results:** The plasma level of miRNA-221 was significantly upregulated in different fibrosis stages of CLD (*p* < 0.05), and this upregulation was positively correlated with the progression of fibrosis (*p* < 0.05). Significantly increased expression of miRNA-221 was found in NAFLD patients compared to HBV patients in the CLD without fibrosis patient group (*p* < 0.05), while expression of miRNA-221 was significantly upregulated among HBV patients in the CLD with the fibrosis group. miRNA-221 showed high diagnostic accuracy in discriminating different stages of fibrosis from healthy control (*p* < 0.05).

**Conclusion:** miRNA-221 may be used as a potential plasma biomarker for early prediction of fibrosis progression in CLD patients.

## 1. Introduction

Chronic liver disease (CLD) is a major cause of morbidity and mortality as well as a considerable burden on medical resources all over the world [1]. Several factors cause CLD including viruses, bacteria, parasites, and noninfectious factors like alcohol, drugs, autoimmune disease, fatty liver, and genetic defects [2]. Globally, 1.5 billion people have CLD, most commonly due to nonalcoholic fatty liver disease (NAFLD) (60%), hepatitis B virus (HBV) (29%), hepatitis C (HCV) (9%), and alcoholic liver disease (ALD) (2%) [3]. The major complications of CLD—cirrhosis and liver cancer—account for 3.5% of all deaths worldwide [1]. In Bangladesh, HBV and NAFLD are the two most common causes of CLD [4]. Invariably, chronic HBV infection or NAFLD causes liver damage that frequently progresses to major liver illnesses including cirrhosis and HCC [5].

MicroRNAs (miRNAs) are small, noncoding, single-strand RNAs of 19–22 nucleotides and have an important role in the regulation of gene expression [6]. Through complementary base-pairing, miRNAs can target mRNAs, causing the posttranscriptional suppression of specific protein-coding genes [7]. More than 2000 miRNAs have been identified to date, and miRNAs are thought to be directly involved in the regulation of up to 60% of all human genes [8]. A rising number of research have shown that miRNAs have a crucial role in CLD progression by regulating proliferation, fibrosis, and apoptosis as well as in carcinogenesis [9]. At the molecular level, the fate of hepatic stellate cells (HSCs) in the fibrotic liver is influenced by a range of miRNAs, which may be used to diagnose fibrosis and to help target antifibrotic medications [10]. Among the wide variety of miRNAs, significant role of miRNA-221 in the regulation of fibrotic pathway by directly activating HSC by different molecular mechanisms is reported in various studies [7, 8, 10]. Therefore, miRNAs may be used as a novel class of noninvasive biomarkers because of their stability and presence in almost all body fluids.

However, to the best of our knowledge, no literature reports on the expression of miRNA-221 in CLD due to HBV and NAFLD as well as at different stages of fibrosis in CLD patients from Bangladesh. Therefore, this study is aimed at determining the relative expression of miRNA-221 in the plasma of HBV and NAFLD-induced CLD patients with different stages of hepatic fibrosis. This may help to identify a promising biomarker for differentiating stages of liver fibrosis and future hepatic fibrosis treatment (anti-miRNA antifibrotic drug development) that results from CLD due to HBV and NAFLD.

## 2. Methods

### 2.1. Study Subjects

This cross-sectional study was conducted in 2023 among 75 CLD patients of different stages of fibrosis either due to HBV or NAFLD attending the Hepatology Department of Bangabandhu Sheikh Mujib Medical University (BSMMU) (Ethical Clearance No. BSMMU/2022/6768). The diagnosis of the patients was confirmed by a hepatologists, and their stages of fibrosis were categorized according to the investigation reports (FibroScan) and the guidelines of METAVIR score of fibrosis: F0, no fibrosis; F1, fibrous portal expansion; F2, few bridges or septa; F3, numerous bridges or septa, and F4, cirrhosis [11]. Chronic hepatitis B (CHB) was diagnosed as people who have HBsAg persisting in their blood for 6 months or longer. Cirrhosis was diagnosed as presence of regenerative nodules surrounded by fibrous bands due to chronic liver injury leading to portal hypertension and end-stage liver disease. NAFLD was diagnosed as the entire spectrum off fatty liver disease (FLD) in individuals without significant alcohol consumption, ranging from fatty liver to steatohepatitis (SH) to cirrhosis. NASH-cirrhosis was diagnosed as presence of cirrhosis with current or previous histological evidence of steatosis or SH.

Twenty-five participants were included in each group—CLD without fibrosis (F0) (Group-II), CLD with fibrosis (F1–F3) (Group-III), and CLD with cirrhosis (F4) (Group-IV), and 25 healthy individuals (Group-I) were included as control. Both HBV-induced CLD and NAFLD-induced CLD patients were included in each patient group. A brief demographic data was collected through a predesigned questionnaire.

### 2.2. Laboratory Procedures

Five milliliters of peripheral venous blood was collected from each participant and centrifuged for 10 min at 3000 rpm at 4°C temp, and tubes were preserved at −70°C with proper labelling until further procedure. RNA was extracted from the plasma sample using QIAGEN miRNeasy Serum-Plasma Kit (QIAGEN, Germany). Extraction was followed by the first strand cDNA synthesis as per the instruction of manufacturer (FIREScript cDNA Synthesis Kit, Solis BioDyne, Tartu, Estonia). For gene expression analysis, specific primers were used (Supporting Information (available here)). Fold changes of miRNA were observed by real-time PCR method using QuantStudio 5 (Applied Biosystems, United States) using the 5× HOT FIREPol Eva Green qPCR Mix Plus (ROX) (Solis BioDyne, Tartu, Estonia). As an internal control, U6SnRNA (small nucleolar RNA) was used to normalize the PCR reaction of selected miRNA. According to manufacturer's protocol, viral load of HBV was detected by GeneProof HBV PCR kit on plasma sample. After a successful run, the data was collected setting the threshold value for miRNA-221 and fold changes were calculated by comparative 2-*ΔΔ*CT method [12]. Using the 2-*ΔΔ*CT method (*ΔΔ*CT = [CT target − CT U6snRNA] test group − [CT target − CT U6snRNA] control group), the data were presented as the fold change in miRNA expression normalized to endogenous reference gene U6 and relative to the healthy control.

### 2.3. Statistical Analysis

The statistical analysis of the data was performed using SPSS software Version 25.0 (IBM, New York City, NY). Expression data was presented as median (IQR). The results of differences in the expression of miR-221 among groups were analyzed by Kruskal–Wallis test and between groups was analyzed by test post hoc analysis (Dunn's). The differences of expression in between subgroups were analyzed by the Mann–Whitney test. The correlation of selected miRNA with laboratory parameters among different study groups was analyzed by the Spearman correlation test. Receiver operating characteristics curve (ROC) analysis was performed to evaluate the diagnostic performances of miRNA-221 to discriminate different stages of fibrosis in CLD patients from healthy control.

## 3. Result

The demographic and clinical characteristics of participants (age, gender, biochemical test: ALT, AST, S. albumin, and viral load) are summarized in Table 1. Statistically significant differences of ALT, AST, and S. albumin levels were found when compared among groups (*p* < 0.05).

The level of miRNA-221 was found to be higher in all three patients' groups when compared to healthy control (Table 2). The highest expression was observed in CLD with cirrhosis patients which were statistically significant (*p* < 0.05) than that of healthy control. CLD with fibrosis patients also had significantly higher expression of miRNA-221 when compared to CLD without fibrosis and healthy control (*p* < 0.05) (Table 3).

Fold change increased more in NAFLD patients (3.12 fold) than that of HBV patients (1.86 fold) in the CLD without the fibrosis group, and the difference was found statistically significant (*p* < 0.05) (Table 4). Mean fold change was found significantly higher in CLD with the fibrosis group due to viral cause (HBV) than the same group of patients with nonviral cause (NAFLD) (*p* < 0.05). However, no statistically significant difference was found in the expression of miR-221 between HBV and NAFLD-induced CLD with cirrhosis patients.

**Figure 1:**
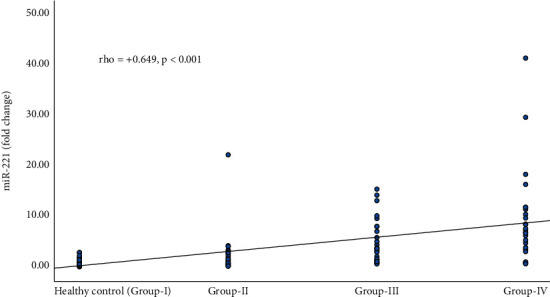
Correlation of expression of miRNA-221 with stages of fibrosis in CLD patients. *p* < 0.05 was considered significant; rho = Spearman correlation coefficient; (+) indicates positive relationship; (−) indicates negative relationship; rho = ±1 indicates perfect correlation. Healthy control (Group-Ⅰ), CLD without fibrosis (F0) (Group-Ⅱ), CLD with fibrosis (F1–F3) (Group-Ⅲ), and CLD with cirrhosis (F4) (Group-Ⅳ).

The expression of miRNA-221 was positively correlated with increasing stages of fibrosis and was statistically significant (*p* < 0.05) (Figure 1).

miRNA-221 expression showed a positive correlation with ALT in CLD without fibrosis (rho = 0.069) and in CLD with cirrhosis (rho = 0.224) which was not statistically significant. However, in CLD with fibrosis, a negative correlation (rho = 0.578) was observed and it was found statistically significant (*p* < 0.05) (Table 5).

ROC analysis was performed to evaluate the diagnostic performances of miRNA-221 to discriminate CLD with different stages of fibrosis patients from healthy control. The diagnostic values of miRNA-221 were determined by the Youden index from the ROC (Table 6).

## 4. Discussion

HBV and NAFLD are the two most common causes of CLD in Bangladesh. Since the progression of CLD varies in patients, distinguishing between rapid from slow fibrosis will allow a better tailoring of treatment. Thus, in terms of preventing liver cirrhosis and end-stage liver disease, early diagnosis and sustained follow-up of the progression of liver fibrosis are essential [13]. miRNAs have been proposed as promising biomarkers for CLD because of their stability and presence in almost all body fluids. A large number of miRNAs have been studied concerning hepatic injury in last decade, and miRNA-221 has already been included in several panels used for liver fibrosis screening [14]. The present study was conducted to observe the expression of miRNA-221 in plasma to find out their relation with stages of CLD patients induced by HBV and NAFLD.

In this study, significant upregulation of miRNA-221 was observed in CLD patients with fibrosis (F1–F3) and CLD patients with cirrhosis (F4) in comparison to that of healthy control (*p* value < 0.05). This upregulation was progressive as stages of CLD progressed. These findings are supported by another study where a significant increase of miRNA-221 in plasma among different stages of CLD patients was observed [15]. They observed a significant increase of miRNA-221 expression in late stages of fibrosis (F3–F4) than that of early stages fibrosis (F1–F2), as well as increased expression found in early stages of fibrosis groups than healthy control. El-Garem et al. [16] showed that miRNA-221 was upregulated in late stages of fibrosis patients than normal patients. Another study demonstrated that the expression of miRNA-221 increases with the progression of human liver fibrosis and is correlated with the expression level of *α*1 (I) collagen and *α* smooth muscle actin mRNAs [17]. Previous several studies reported that miRNA-221 increases fibrosis directly or via modulation of HSC proliferation by inhibiting posttranscriptional target G-protein alpha inhibiting activity polypeptide 2 (*Gnai2*), *c*yclin-dependent kinase inhibitors (CDKNs) such as *CDKN1C* and *CDKN1B*, or regulating hepatocyte apoptosis and proliferation by targeting p53 upregulated modulator of apoptosis (*Puma*) and aryl hydrocarbon receptor nuclear translocator (*Arnt)* and blocking its function in hepatocytes [18–20]. In the present study, the progressive upregulation of miRNA-221 may be due to these mechanisms. On the contrary, Appourchaux et al. claimed that there were no significant differences in the expression of miRNA-221 between late-stage fibrosis (F3–F4) and early-stage fibrosis (F1–F2) in CHB patients [21]. The present study revealed a significant difference in the expression of miRNA-221 between HBV and NAFLD subgroups in CLD without fibrosis and CLD with fibrosis (*p* < 0.05). Several studies had reported the role of miRNA-221 in different stages of fibrosis in CHB and NASH-induced liver fibrosis [2, 13, 22, 23], but no previous study was found where expression of miRNA-221 between HBV and NAFLD patients was analyzed in CLD patients based on fibrosis stages. However, one previous study reported increased expression of miRNA-221 in advanced stages of fibrosis [17] and no difference between early and late stages of fibrosis in another study [21]. The disparity in the expression of miRNA-221 between HBV and NAFLD patients may be due to disease etiology and small number of study participant in the subgroups. Further study is needed to validate these findings of the present study. ROC curve analysis was performed to evaluate the accuracy of individual miRNAs under the study to identify the different stages of CLD. The ROC curve revealed that miRNA-221 expression levels could be used with good sensitivity and specificity in the diagnosis of CLD without fibrosis patients (sensitivity, 96%; specificity, 84%), CLD with fibrosis patients (sensitivity, 88%; specificity, 80%), and CLD with cirrhosis (sensitivity, 84%; specificity, 64%) patients. Therefore, the expression level of miRNA-221 may be utilized as an effective noninvasive biomarker in plasma for early detection of CLD due to HBV and NAFLD.

### 4.1. Limitations

There are some limitations of this study. Liver biopsies were not performed among study participants. The smaller number of study participants in subgroups may limit the ability to detect significant differences in the expression of miRNA-221 between HBV and NAFLD patients; therefore, further studies are needed.

### 4.2. Conclusion

The progressive upregulation of miRNA-221 was correlated with fibrosis progression of CLD patients. Thus, it can be used as a potential biomarker for discriminating different stages of fibrosis in CLD patients. Larger cohorts are required to validate the utility of plasma miRNA-221 in monitoring liver fibrosis prognosis. This study will give insight to future researchers in the context of anti-miRNA and antifibrotic drug development and help physicians to scale up interventions to halt fibrosis progression in CLD patients.

## Figures and Tables

**Table 1:** The demographic and clinical characteristics of participants.

**Variables**	**CLD without fibrosis (** **n** = 25**)**	**CLD with fibrosis (** **n** = 25**)**	**CLD with cirrhosis (** **n** = 25**)**
Age group (mean ± SD)	37.4 ± 12.9	37.4 ± 12.9	53.4 ± 10.8
Gender (*n*)			
Male	19	8	17
Female	6	17	8
ALT (U/L)^†^ median (min–max)	52 (12–97)	42 (12–78)	44 (14–622)^∗^
AST (U/L)^†^ median (min–max)	33 (24–92)	40 (24–60)	64 (11–375)^∗^
S. albumin (g/L)^†^	40 (25–49)	40 (32–53)	24 (13–42.1)^∗^
Viral load (log10 copies/mL)^‡^mean ± SD	3.37 ± 0.51	3.24 ± 0.35	4.76 ± 0.51^ҩ^

*Note:* Data were expressed median (min–max) and mean ± SD.

^†^ *p* value obtained by Kruskal–Wallis test.

^ҩ^ *p* value obtained by ANOVA test.

^‡^ *p* value obtained in CLD due to the HBV subgroup.

^∗^ *p* value < 0.05 was considered significant.

**Table 2:** Comparison of fold changes of expression of miRNA-221 in plasma among study groups.

**Study groups**	**Fold change (median [IQR])** ^‡^	**p** ** value**
Healthy control	0.65 (0.37–1.43)	< 0.001^∗^
CLD without fibrosis	1.10 (0.29–2.77)	
CLD with fibrosis	3.63 (1.44–7.62)	
CLD with cirrhosis	7.68 (3.32–11.89)	

Abbreviations: IQR = interquartile range, miRNA = microRNA.

^‡^Kruskal–Wallis test was performed among groups.

^∗^ *p* <0.05 was considered significant.

**Table 3:** Comparison of fold changes of expression of miRNA-221 in plasma between study groups.

**Comparison between groups** ^†^	**p** ** value**
Group II vs. Group III	0.020^∗^
Group II vs. Group IV	< 0.001^∗^
Group II vs. Group I	0.737
Group III vs. Group IV	0.999
Group III vs. Group I	< 0.001^∗^
Group IV vs. Group I	< 0.001^∗^

Abbreviations: Group I = healthy control, Group II = CLD without fibrosis, Group III = CLD with fibrosis, Group IV = CLD with cirrhosis.

^†^Post hoc (Dunn's) test was performed between groups.

^∗^ *p* < 0.05 was considered significant.

**Table 4:** Expression of miRNA-221 in CLD patients between HBV and NAFLD subgroups.

**miR**	**Group**	**Subgroup (** **n** **)**	**Fold changes**		**p** ** value**
			**HBV**	**NAFLD**	
miR-221	CLD without fibrosis	HBV (13) NAFLD (12)	1.86 (1.04–4.00)	3.12 (1.20–3.63)	0.03^∗^
	CLD with fibrosis	HBV (10) NAFLD (15)	6.86 (5.31–10.63)	1.05 (0.27–2.15)	< 0.001^∗^
	CLD with cirrhosis	HBV (14) NAFLD (11)	7.68 (2.61–8.86)	8.63 (4.83–15.28)	0.88

*Note:* Data were expressed as median (IQR). *p* value was obtained by Mann–Whitney test.

Abbreviations: IQR = interquartile range, miRNA/miR = microRNA.

^∗^ *p* < 0.05 was considered significant.

**Table 5:** Correlation of expression of miRNA-221 with ALT, AST, S. albumin, and viral load in CLD patients.

**miRNA**	**Variable**	**Study groups**	**Rho-value**	**p** ** value**
miRNA-221	ALT (IU/L)	CLD without fibrosis	+0.069	0.742
		CLD with fibrosis	−0.578^∗^	0.002
		CLD with cirrhosis	+0.224	0.282
	AST (IU/L)	CLD without fibrosis	+0.025^∗^	0.025
		CLD with fibrosis	−0.305	0.138
		CLD with cirrhosis	+0.082	0.698
	S. albumin (g/L)	CLD without fibrosis	+0.188	0.369
		CLD with fibrosis	−0.144	0.492
		CLD with cirrhosis	+0.158	0.449
	Viral load (log10 copies/mL)	CLD without fibrosis	−0.679	0.094
		CLD with fibrosis	−0.143	0.787
		CLD with cirrhosis	−0.350	0.356

*Note:* (+) indicates positive relationship; (−) indicates negative relationship; rho = ± 1 indicates perfect correlation, rho = ± 0.80 to ± 1 means strong correlation, rho = ± 0.50 to ± 0.80 means moderate correlation, rho = ± 0.20 to ± 0.50 means weak correlation, and rho = ± 0.01 to 0.20 means negligible correlation.

Abbreviation: rho = spearman correlation coefficient.

^∗^ *p* value of < 0.05 was considered as significant.

**Table 6:** Diagnostic performance of miRNA-221 in differentiating different stages of fibrosis in CLD patients from healthy control.

**miRNA**	**Groups**	**AUC**	**95% CI**	**p** ** value**	**Sensitivity**	**Specificity**	**Cutoff value**
miRNA-221	CLD without fibrosis	0.931	0.861–1.00	< 0.001	96%	84%	2.87
	CLD with fibrosis	0.856	0.744–0.968	< 0.001	88%	80%	3.56
	CLD with cirrhosis	0.656	0.501–0.811	0.05	84%	64%	9.79

## Data Availability

The data that support the findings of this study are available from the corresponding author upon reasonable request.
