# Supplementary material for: miRNA-221: A Potential Biomarker of Progressive Liver Injury in Chronic Liver Disease (CLD) due to Hepatitis B Virus (HBV) and Nonalcoholic Fatty Liver Disease (NAFLD)
Source: Int J Hepatol. 2024 Aug 16;2024:4221368. doi: 10.1155/2024/4221368 (PMC11343628; doi:10.1155/2024/4221368)
Supplement: Supporting Information — Additional supporting information can be found online in the Supporting Information section. Primers used in the study. [file 4221368.f1.docx]

**Supplementary files:**

**Primers used in the study**

**miR-221:**

FP- TCGGCGTCCCTGAGACCCTAAC;

RP- GTCGTATCCAGTGCAGGGTCCGAGGT;

RT-GTCGTATCCAGTGCAGGGTCCGAGGTATTCGCACTGGATACGACTCACAA;

**U6:**

FP**-**CTCGCTTCGGCAGCACA**,**

RP-AACGCTTCACGAATTTGCGT,

RT- CTCAACTGGTGTCGTGGAGTCGGCAATTCAGTTGAGAAAAATATG
